# Supplementary material for: Dyslipidemia and associated risk factors among HIV/AIDS patients on HAART in Asmara, Eritrea
Source: PLoS One. 2022 Jul 1;17(7):e0270838. doi: 10.1371/journal.pone.0270838 (PMC9249179; doi:10.1371/journal.pone.0270838)
Supplement: S1 Appendix — (PDF) [file pone.0270838.s001.pdf]

Appendix I: Letter of Introduction & Consent

Asmara College of Health Sciences

Asmara, Eritrea.

Code: _________________

RE: DATA COLLECTION

Dear Respondent,

We are students at the College of Health Sciences (ACHS). We are currently doing a research titled, Lipid Abnormalities and Associated Risk Factors among HIV/AIDS Patients on HAART in Asmara, Eritrea.

You have been selected to participate in this study and we would highly appreciate if you assisted us by responding to all questions as completely, correctly and honestly as possible. Your response will be treated with utmost confidentiality and will be used only for research purposes of this study only.

Thank you in advance for your co-operation.

Yours Faithfully,

Signature: _____________ Date: ______________________

Appendix II: Study Questionnair

ASMARA COLLEGE OF HEALTH SCIENCES

Independent project; Frequency of dyslipidemia and correlates in HIV/AIDS patients on HAART in Asmara, Eritrea

Introduction

We are carrying out an academic research on lipid abnormalities and associated risk factors among HIV/AIDS patients on HAART in two Referral Hospitals in Asmara, Eritrea. Kindly spare some few minutes of your time to fill this questionnaire. Please respond to the following questions and where applicable. The responses you provide will be strictly confidential. No reference will be made to any individual(s) in the report of the study.

<u>Questionnaire Form</u>

Date: _____________

Patient identification code_____________

Patient card number _______________

S.N	**I. Sociodemographic data**		
1.	Age		
2.	Gender	1. Male 2. Female	
3.	Educational level	1. Illiterate 2. Elementary<br>3. Junior 4. Secondary<br>5. College and above	
4.	Ethnic group		
5.	Area of residence		
	**II. Anthropometric data**		
1.	Weight in kilogram		
2.	Height in meters		
3.	Calculated BMI		
4.	Hip to Waist ratio	Hip ______<br>Waist ______	

5.	Physical appearance i.e Obesity or fat accumulation in any part of the body		
	**III. Line of treatment**		
1.	Drug combination		
2.	Duration of HAART exposure to the current drug		
3.	Reported side effect		
4.	Previous drug and duration of exposure to that drug		
5.	Reported side effect		
	**IV. Medical and clinical history**		
1.	Are you taking any other drugs?	1. Yes 2. no	If no skip to no. 3
2.	What are these drugs?		
3.	Do you have any cardiovascular disease (CVD)?	1. Yes 2. no	If no skip to no 5
4	Specify the type of CVD		
5.	Have you ever had hypertension?	1.Yes 2.No	
6.	Does any member of your family have CVD?	1.Yes 2.No	
7.	CD-4 count		
	CD-8 count		
8.	viral load result		

	**V. Life style**		
1.	Do you do any physical exercise?	1. Yes 2. No	If no go to no 3
2.	How often do you do physical exercise?		
3.	Do you eat fat containing foods?	1.Yes 2.No	If no go to no 5

4.	How often do you eat fat containing foods?		
5.	Do you smoke?	1. Yes 2.No	If no go to no 7
6.	How many cigarettes per day do you smoke?		
7.	Do you drink alcohol?	1. Yes 2. No	If yes go to no 8
8.	What type of alcohol do you drink?	1.Sewa 2.Beer 3.Whisky 4.Wine 5.other	
9.	How often do you drink alcohol?		

Thank you in advance for your cooperation!

Your signature______________
