# Supplementary material for: Dyslipidemia and associated risk factors among HIV/AIDS patients on HAART in Asmara, Eritrea
Source: PLoS One. 2022 Jul 1;17(7):e0270838. doi: 10.1371/journal.pone.0270838 (PMC9249179; doi:10.1371/journal.pone.0270838)
Supplement: Attachment — Submitted filename: Responses to reviewers.pdf [file pone.0270838.s003.pdf]

Rebuttal Letter: Response to Reviewers

Reviewer 1

1. *Abbreviation issues: Acronyms like HAART, LDL, TC, CVD, etc… should be fully written on their first appearance. DONE.*

   - All acronyms that has been mentioned in the study are written in full in their first mention.

2. *In the results section, CD4 should be corrected as "CD4+ T cell count (cell/microliter)".*

   - "CD4" has now been corrected as "*CD4+ T cell count (cell/microliter)*" in the revised manuscript.

3. *Results with a significant association at aOR should be considered the real predictors not cOR data.*
   - General frequencies and the key risk factors which have showed significant association in multivariate regression analysis are indicated in the abstract section. Similar approach has also implemented in the conclusion section.

4. *In the results, the cOR values better be displayed for readers convenience.*

   - Univariate regression analysis table is now included in the manuscript.

5. *In the conclusion, factors significantly associated with dyslipidemia should be highlighted.*

   - The main risk factors that have been revealed statistically are outlined in the conclusion section. Necessary interventional recommendation have also been included.

6. *Subtitles in the discussion could be removed.*
   - The discussion section is now summarized under one title detailing about all the prevalence, effect of drug regimens and factors associated with dyslipidemia.

7. *The limitations of the study should be stated at the last paragraph of the discussion. Then the importance of the study can be mentions.*

    - The last paragraph of the discussion section is rewritten according the reviewers guide.

8. *The conclusion should be a single paragraph without result values like percentages and showing burden of dyslipidemia in other means of expression.*

    - The conclusion is formatted now in one paragraph showing the key findings of the study and other important predictors. A brief recommendation is also included in the last lines of the paragraph.

Reviewer 2

9. *There are long and repetitive sections within the methods with not important data.*

    - The methods part is now summarized and details on inclusion criteria, data collection have been revised to indicate the key information regarding study participants.

10. *At what time of HAART therapy were the patients evaluated for lipid profile?*

- At the time of blood sampling, the duration of HAART use in the patients ranged from 1 year to 16 years. The mean duration of HAART use was 4.57(±2.43) years. The prevalence of dyslipidemia worsens across time after ART initiation, despite initial favorable changes in TG and HDL during the 6 months period. To minimize this apparent effect in this study, only patients who had HAART for more than one year were included.
- This has been explained in the second paragraph of the results portion.

11. *Do we have baseline or pre-therapy values in at least some of the patients?*

- Due to the cross sectional nature of the study, lipid profile assessments were conducted during the therapy time. Currently, evaluation of fat levels in HIV patients in Eritrea is not a regular package of the pre-therapy investigations like CD4 count, viral load, kidney function and blood sugar. Some few patients may have lipid profile tests during first stages of therapy period by specific physician order to evaluate complications related to the disease.
- One of the aim of this study is to include lipid profile tests in all HIV patients immediately before the initiation of HAART treatment and to sustain its continuous examination during therapy. This have been outlined at the last paragraph of the conclusion portion.

12. The therapy taken by patients should be better analyzed, looking for the use of statins in at least part of them.

- All therapies including accessory drugs taken by patients for other complications related to the disease were assessed. Patients who were taking any lipid altering or anti-dyslipidemic drugs were primarily excluded from the study. These include statins like, but not limited to, atorvastatin, fluvastatin, lovastatin and rosuvastatin which are commonly prescribed in the study setting. Moreover, patients who were generally taking fibrates, bile acid sequestrants, niacin and ezetimibe were also excluded for their effect in altering lipid values in patients.
- This has been indicated at the sampling technique and inclusion criteria subtitles of the methodology portion.

13. *The text is very difficult to read, especially the presentation of the results. A complete revision is needed by native speakers. There are also widespread grammatical errors and typos.*
- The manuscript have been revised for spelling and typing errors. Moreover, the text have been overviewed by two English native speakers for grammatical issues. Portions which were not clear have been corrected in the revised manuscript.
