# Supplementary material for: Dyslipidemia and associated risk factors among HIV/AIDS patients on HAART in Asmara, Eritrea
Source: PLoS One. 2022 Jul 1;17(7):e0270838. doi: 10.1371/journal.pone.0270838 (PMC9249179; doi:10.1371/journal.pone.0270838)
Supplement: Attachment — Submitted filename: Review comments.docx [file pone.0270838.s002.docx]

**Review comments**

Title: Lipid abnormalities and associated risk factors among HIV/AIDS patients on HAART in Asmara, Eritrea

Submission ID: PONE-D-22-02948

The authors assess dyslipidemia associated with HAART in HIV/AIDS patients which an important topic to uncover the negative impacts of the treatment. So far, many studies elucidated this scenario and I appreciate the novelty of this study conducted in Eritrea for the first time. The point by point concerns are detailed below.

**Title:** the title better be “Dyslipidemia…..

**Abstract**:

Acronyms like HAART, LDL, TC, CVD, etc… should be fully written on their first appearance

In the results section, CD4 should be corrected as “CD4+ T cell count (cell/microliter)”. In addition, results with a significant association at aOR should be considered the real predictors. cOR data cannot be inferred or reported as final outcome of association

In the conclusion factors significantly associated with dyslipidemia should be highlighted

**Results**

In the results, the cOR values better be displayed for readers convenience

**Discussion**

Subtitles in the discussion could be removed. Besides, the limitations of the study should be stated at the last paragraph of the discussion. Then the importance of the study can be mentions

**Conclusion**

The conclusion should not contain results values like percentages. The conclusion should be a single paragraph containing the burden of dyslipidemia (not in percentages, rather saying either “significantly high” or other means of expression). Then important predictors are mentioned and lastly a brief recommendation should be included.
